# The effect of self-made video assessment on standardized nursing skills training

**DOI:** 10.3389/fmed.2025.1705526

**Published:** 2026-01-14

**Authors:** Yanran Li, Hui Zhao, Bo Zhang

**Affiliations:** 1 Department of Prevention and Health Care, Xingtai People’s Hospital, Xingtai, Hebei, China; 2 Nursing Department, Xingtai People’s Hospital, Xingtai, Hebei, China; 3 Department of Pancreaticobiliary Surgery, Xingtai People’s Hospital, Xingtai, Hebei, China

**Keywords:** assessment mode, nurses, nursing education, self-made video, standardized skills training

## Abstract

**Objective:**

This study aimed to explore the effect of a self-made video assessment mode in standardized skills training of nurses.

**Methods:**

This study adopted a randomized controlled trial design. A total of 110 new female nurses who came to our hospital from January 2020 to December 2020 to participate in standardized training for new nurses were selected as the study objects. They were randomly divided into the control group (CG) and study group (SG). The new nurses of the two groups were trained in a unified way by the specialist head nurse. After the training, the new nurses of the CG were assessed in a traditional manner. The new nurses in the SG were evaluated by self-video recording. The basic theory and basic operation assessment results, core competence, self-efficacy, practice time, and satisfaction of nurses in two groups were compared.

**Results:**

In contrast to the CG, the basic theory and basic operation assessment results in the SG presented elevation (*p* < 0.05), the CIRN score and GSES score in the SG presented elevation (*p* < 0.05), the practice time of nurses in the SG presented longer (*p* < 0.05), and the satisfaction of nurses in the SG presented elevation (*p* < 0.05).

**Conclusion:**

Self-made video assessment mode can promote the standardized skills, core competence, and self-efficacy of nurses in standardized skills training of nurses.

## Introduction

The standardized training of new nurses, which is a structured and systematic program aimed at enabling novice nurses to acquire the necessary clinical skills and professional qualities by following a curriculum consistent with national medical standards, is an important part of the standardized training for national health technicians and is also a crucial measure for ensuring the development of the nursing profession (1). New nurses constitute the emerging workforce of the clinical nursing team (2). Systematic and standardized training not only lays the foundation for the selection and cultivation of high-quality nursing talents but also directly affects patient safety and the comfort of hospitalized patients (3). However, although its importance is self-evident, the effectiveness of standardized training is limited by the inefficiency of the assessment methods. How to optimize this training through innovative assessment methods remains a focus of discussion among various medical institutions (4).

Currently, hospitals have increasingly higher requirements for the comprehensive abilities of graduating nurses and nursing students before their internships (5). The survey indicates that the clinical environment demands that newly hired nurses possess basic clinical skills and comprehensive humanistic qualities to meet the constantly changing medical needs (6). Therefore, it is crucial to cultivate these abilities through structured training programs (7). The traditional assessment method—where multiple teachers supervise the skill examination simultaneously—consumes a lot of resources, is prone to subjective bias, and cannot fully reflect the students’ true abilities due to the candidates’ nervousness (8). Moreover, expanding the enrollment scale and increased clinical workload of teachers make personalized practical assessment difficult, exacerbating the gap in training quality control (9).

Although there is a growing recognition of the need for solutions that can balance the strictness of assessment and resource efficiency to improve nursing education, the existing research in this specific field is relatively limited. While some studies have explored various technology-based assessment tools used in healthcare and medical education, overall, the number of such studies is still insufficient. For example, Knaff et al. studied the application of virtual simulation technology in the assessment of neurosurgical technical skills (10). Sahadevan et al. explored the effectiveness of online assessment platforms in medical training (11). However, in the realm of standardized nursing training, there are still significant and comprehensive research gaps. Specifically, there is a lack of specialized, scalable, and technology-based solutions that can effectively balance the strictness of assessment and resource efficiency. This gap in the literature is the specific area that this research aims to fill. Existing research has not adequately addressed the unique challenges faced in standardized nursing training assessment, such as the high resource demands of traditional methods and the need for more objective and student-centered approaches. This highlights the novelty and potential importance of this study in meeting this unmet need.

Self-made video recording assessment is a novel assessment method, where students use multimedia tools (such as cameras and digital recorders) to record their performance of skills for subsequent review by teachers (12, 13). This method overcomes the time and space limitations of traditional face-to-face assessment, reduces the pressure brought by examinations, and minimizes potential psychological biases that may affect the results (14, 15). By leveraging the existing digital infrastructure, this study meets the demand for an economically efficient, standardized, student-centered, and in line with modern nursing education needs assessment tool.

This study aimed to evaluate the effectiveness of self-made video assessment in enhancing the standardized training outcomes for nurses, with the specific goal of filling the aforementioned gap in the literature by providing a specialized, scalable, and technology-based solution for standardized nursing training assessment.

## Data and methods

### Study design

This study adopted a randomized controlled trial design. A total of 110 new female nurses who came to our hospital from January 2020 to December 2020 to participate in standardized training for new nurses were selected as the study objects. Inclusion criteria: (1) Nurses who passed the assessment within 2 years of entry; (2) Nurses willing to cooperate with research. Exclusion criteria: (1) Nurses who had participated in similar training; (2) Nurses unwilling to cooperate with the research. This study was approved by the ethics committee of the People’s Hospital of Xingtai, and all nurses signed the informed consent form.

### Randomization

To ensure that the two groups of research subjects were comparable at the baseline level and to reduce selection bias, a simple random grouping method was adopted for the grouping. The specific operation was as follows: First, 110 newly recruited female nurses were numbered according to the order of admission, from 1 to 110. Then, using the random number table method, an initial number and an increasing direction from the random number table were randomly selected, and two-digit random numbers were read successively. For each randomly read number, if its value is within the range of 1–55, the corresponding numbered nurse was assigned to the control group (CG); if its value is within the range of 56–110, the corresponding numbered nurse was assigned to the study group (SG). If a duplicate random number occurred, it was skipped, and the next number was read until all 110 nurses were grouped. Finally, each group would include 55 nurses.

### Blinding

To minimize potential bias and ensure the internal validity of the study, we implemented blinding procedures. The participants were informed that they would be participating in a study comparing different assessment methods for standardized skills training, but they were not explicitly informed about their specific group assignment to prevent any potential performance bias based on group awareness.

The assessors were also blinded to the group allocation status. For the traditional assessment in the CG, the assessors were not aware of which group the nurses belonged to when conducting the evaluations. For the self-video recording assessment in the SG, the videos were anonymized before being presented to the assessors. The assessors only evaluated the skills demonstrated in the videos without any knowledge of whether the nurse was from the SG or CG, thus reducing the risk of subjective bias in the assessment process.

### Baseline data collection

Baseline data, including age and educational background, were gathered through a structured questionnaire. The questionnaire was designed by a panel of experienced nursing educators and researchers, ensuring its validity and reliability. Before the start of the standardized training program, the new nurses were invited to fill out the questionnaire in a quiet and private environment. To ensure the accuracy of the data, the researchers were available on-site to answer any questions and provide clarification if needed. For age data, the nurses were asked to report their exact birth dates, and the age was then calculated based on the current date. Regarding educational background, the nurses were required to select from options, including college, bachelor’s, and master’s, according to their highest level of completed education. All the collected data were double-entered into a secure electronic database by two independent researchers to minimize data entry errors.

## Methods

The new nurses in both groups were uniformly trained by the departmental nurse managers. The selected nurse managers all possess rich clinical nursing experience, solid professional knowledge, and excellent teaching abilities. Their working years in related nursing fields are all over 10 years, and they have received systematic teacher training, enabling them to proficiently apply various teaching methods in the training process. The total duration of this standardized nurse training program was 6 months. For each month, there are 4 training sessions, and each session lasts for 2 h. The cumulative training duration reaches 48 h. This schedule was designed to ensure that new nurses have sufficient time for systematic theoretical learning and practical operation training, gradually mastering various standardized nursing skills.

The training content included: (1) Basic theoretical knowledge, such as nursing ethics and law, fundamentals of human anatomy and physiology, and common disease care knowledge; (2) Nursing operation skills, including basic nursing procedures and practical nursing procedures; (3) Communication and humanistic care skills, including communication skills between nurses and patients and concept and practice of humanistic care.

The training goals included: (1) Knowledge and skill goals: Through training, new nurses can comprehensively master the basic theoretical knowledge of nursing and the knowledge of common diseases, and be able to accurately apply the learned knowledge to analyze and solve clinical nursing problems. They can also master various basic nursing operations and specialized nursing operation skills, achieving a level of standardized, proficient, and accurate operation, to ensure the safety of patient care. (2) Attitude and value goals: Through training, new nurses can cultivate good professional ethics and attitudes, establish a patient-centered service concept, and enhance their sense of responsibility and mission. They can also improve their teamwork spirit and communication skills, enabling them to cooperate effectively with doctors, nurses, and other healthcare professionals to provide high-quality nursing services for patients. (3) Achievement goals for skills development: Through training, new nurses can enhance their clinical thinking skills and problem-solving abilities, enabling them to make correct judgments and decisions promptly in complex clinical situations. They can also develop their self-learning abilities and innovative thinking skills, and be encouraged to continuously explore and improve their nursing methods, thereby improving the quality of nursing care.

Material requested: (1) Textbooks and materials: Authoritative nursing professional textbooks were selected as the main reference materials for the training, such as “Fundamental Nursing,” “Nursing in Internal Medicine,” and “Nursing in Surgery.” At the same time, internal hospital nursing operation norms, clinical case collections, and nursing quality management documents were collected and organized for new nurses to study and refer to. (2) Teaching equipment and materials: Complete sets of teaching equipment and materials were provided, including manikins, nursing operation models (such as injection models, catheterization models, cardiopulmonary resuscitation models), electrocardiogram monitors, defibrillators, nebulizers, and infusion pumps, to offer new nurses a realistic operating environment and practice opportunities. (3) Multimedia teaching resources: Multimedia teaching courseware was created using images, videos, and animations to present the nursing operation procedures and clinical cases, making the training content more vivid and understandable. At the same time, by utilizing online learning platforms, new nurses were provided with abundant learning resources, such as nursing lecture videos, online tests, and discussion areas, facilitating their self-study and communication.

The chief nurse of the specialized department conducted the training in accordance with the training plan and content, using a combination of various teaching methods such as theoretical lectures, practical operation demonstrations, group discussions, case analyses, and simulation exercises. During the training process, emphasis was placed on interaction and communication with new nurses, timely answering their questions, and adjusting the training progress and methods based on the learning situation and feedback of the new nurses to ensure the training effectiveness.

After the training, the new nurses in the CG were assessed using the traditional way, which included a professional evaluation by the training head nurses. The nurses were organized to attend specialized hospital departments for a unified assessment. They were then arranged according to the first character of their names and demonstrated the nursing skills they had learned one by one according to their assigned serial numbers.

After the training, the new nurses in the SG were evaluated by self-video recording. New nurses recorded an operation video and submitted it to the e-mail of professional assessment teachers 1 week after the end of training, including pre-operation assessment, preparation, implementation, and evaluation. The video picture was clear and stable, the light was suitable, the main body was clear, and the sound was clear.

Before conducting the self-video assessment, the new nurses were organized to attend a special guidance session. During the session, the purpose, process, and importance of the self-video assessment were explained in detail to the new nurses, and the influence of video quality on the assessment results was emphasized. To ensure that new nurses fully understand the requirements for video recording, a written “Self-made Video Recording Guidance Manual” was provided, covering the following aspects: (1) New nurses should complete the recording of operation videos within 1 week after the training to ensure that the assessment content reflects their recent training achievements and avoids the impact on assessment accuracy due to a long-time interval. (2) The videos should cover all the operation steps, including preoperative assessment (such as patient’s physical condition assessment and psychological state assessment), preparation (item preparation and environment preparation), implementation (specific operation steps and techniques), and assessment (post-operation effect assessment and patient feedback collection), ensuring that new nurses fully demonstrate the standardized skills they have learned during the training. (3) Specific standards are set for the video picture quality, requiring clear and stable images, avoiding blurry or shaky situations; appropriate lighting to ensure that the operation process can be clearly presented without shadows or overly bright areas that affect observation; the subject should be clear, with the new nurses’ operation actions and the items used being in the center of the picture and easily identifiable; the sound should be clear and distinguishable, ensuring that the assessment teachers can accurately hear the explanations, exchanges, and environmental sounds during the operation process, so as to conduct a comprehensive assessment. At the guidance meeting, the new nurses were provided with a detailed introduction to the standards and evaluation criteria on which the assessment is based, and the “Explanation Document of Assessment Standards and Evaluation Criteria” was distributed. This document assesses the operational skills of nurses from multiple aspects, as follows: (1) According to the standardized operation procedures, the accuracy of each operation step is scored. (2) The smoothness and speed of the new nurse’s operation are observed. Proficient operation should be manifested as continuous and natural movements, without obvious pauses or hesitations, and the ability to complete the operation within a reasonable time. (3) The communication situation between the new nurse and the patient or other medical staff during the operation is assessed. This includes whether information was clearly and accurately conveyed, whether the patient’s demands could be patiently listened to and appropriate responses given, and whether the communication tone was friendly and gentle. (4) Some possible emergencies are simulated, and whether the new nurse can promptly identify and take correct response measures in the video is observed, and their emergency response speed and problem-solving ability are evaluated. (5) Factors such as the video’s picture quality, sound quality, and content completeness are considered. Picture and sound quality directly affect the assessment teacher’s observation and understanding of the operation process, while content completeness ensures that the assessment can comprehensively cover the required skill points.

### Observation indicators

1. Assessment results: After 6 months of training for the two groups, basic theory and basic operation assessments were carried out uniformly. The basic theory examination used a questionnaire format, in which all theoretical questions were randomly selected from the question bank. The type and number of questions were identical for all participants, with a full score of 100. The basic skills assessment questions were based on the training received by the new nurses. During the first 15 min of the assessment, the new nurses drew lots to determine their assessment questions. The full score was 100.
2. After the training of two groups of nurses, the core competence of the nurses was assessed by Competency Inventory for Registered Nurses (CIRN) (16), which consisted of 58 items in 7 dimensions. The total score ranged from 0 to 232, and the higher the score, the stronger the core competence of nurses. Regarding the cultural adaptation and validation of the CIRN, we first conducted a thorough literature review to understand its original development context and theoretical basis. Given that our study was conducted in a different cultural and clinical setting, we organized a panel of bilingual nursing experts, including those with experience in international nursing research and local clinical practice. This panel reviewed each item of the CIRN to assess its cultural relevance and appropriateness. Items that were deemed potentially culturally insensitive or not applicable were either modified or replaced with more contextually relevant alternatives. After the initial adaptation, we conducted a pilot test with a small sample of 30 local nurses to gather preliminary data on the scale’s performance. Based on the pilot test results, we further refined the items to ensure clarity and comprehensibility. Finally, we calculated the Cronbach’s alpha coefficient for the adapted CIRN, which was 0.87, indicating good internal consistency in our cultural context.
3. After the training of two groups of nurses, the self-efficacy of the nurses was assessed by the general self-efficacy scale (GSES) (17), with a total of 10 items, ranging from completely incorrect to completely correct, ranging from 1 to 4 points, with the score ranging from 10 to 40 points. The higher the score, the better the self-efficacy was. For the GSES, we followed a similar cultural adaptation and validation process. We started by reviewing the original scale and its psychometric properties. Then, we engaged the same panel of bilingual nursing experts to evaluate the cultural appropriateness of each item. Some items were slightly reworded to better fit the local cultural norms and language usage. After the adaptation, we administered the scale to a sample of 35 local nurses for a pilot study. The data from the pilot study were analyzed to check for any issues with item interpretation or scale performance. Based on the findings, we made final adjustments to the scale. The Cronbach’s alpha coefficient for the adapted GSES was 0.91, demonstrating high internal consistency in our study setting.
4. The practice time of the two groups after training was compared.
5. A self-developed satisfaction questionnaire was used to evaluate nurses’ perceptions of their respective training modes. The questionnaire was designed based on a literature review of nursing training satisfaction scales and input from clinical nursing experts. In the process of literature review, we referred to the study by Arkan et al. (18), which presented a well-established nursing training satisfaction scale. This scale provided a solid foundation for our self-developed questionnaire. We carefully analyzed its item structure, content domains, and scoring system, and adapted relevant elements to fit the specific context of our study on standardized skills training for new nurses.

In terms of cultural adaptation and validation for this self-developed satisfaction questionnaire, we first identified key domains and items from the existing literature and the Arkan et al. scale that were relevant to our study context. These items were then translated into the local language and back-translated by a professional translator to ensure accuracy. Next, we organized focus group discussions with local nurses and nursing educators to gather their feedback on the relevance and clarity of the items. Based on their input, we revised the items to better reflect the local nursing training environment and cultural expectations. After that, we conducted a pre-test on a small sample of 20 new nurses who had similar characteristics to the study participants. The Cronbach’s alpha coefficient was calculated for the overall questionnaire and each domain. The overall Cronbach’s alpha was 0.85, indicating good internal consistency. The Cronbach’s alpha coefficients for the training content, teaching methods, and overall experience domains were 0.80, 0.78, and 0.82, respectively, suggesting acceptable to good reliability within each domain.

This questionnaire included 10 items covering three domains: training content (4 items), teaching methods (3 items), and overall experience (3 items). Each item was scored on a 10-point Likert scale (1 = “very dissatisfied” to 10 = “very satisfied”), with a total possible score of 100. Very satisfied: total score > 90; satisfied: total score 70–89; dissatisfied: total score < 70. Nursing satisfaction was calculated as the percentage of nurses who scored “very satisfied” or “satisfied” relative to the total number of participants in each group.

### Statistical analysis

SPSS 20.0 statistical software was adopted for statistical analysis of the data. Measurement data were expressed as (x ± s), and the *t*-test was adopted for comparison. Statistical data were exhibited as [n (%)] and were measured using the χ^2^-test. *p* < 0.05 was considered statistically significant.

## Results

### Baseline data between the two groups

Nurses in the CG were aged 22–36 years, with an average age of 29.28 ± 3.02 years. There were 40 cases of college, 10 cases of bachelor’s, and 5 cases of master’s. Nurses in the SG were aged 23–37 years, with an average age of 29.58 ± 3.08 years. There were 41 cases of college, 10 cases of bachelor’s, and 4 cases of master’s. No difference was discovered in general data between the two groups (*p* > 0.05), indicating comparability.

### Assessment results between the two groups

**Figure 1:**
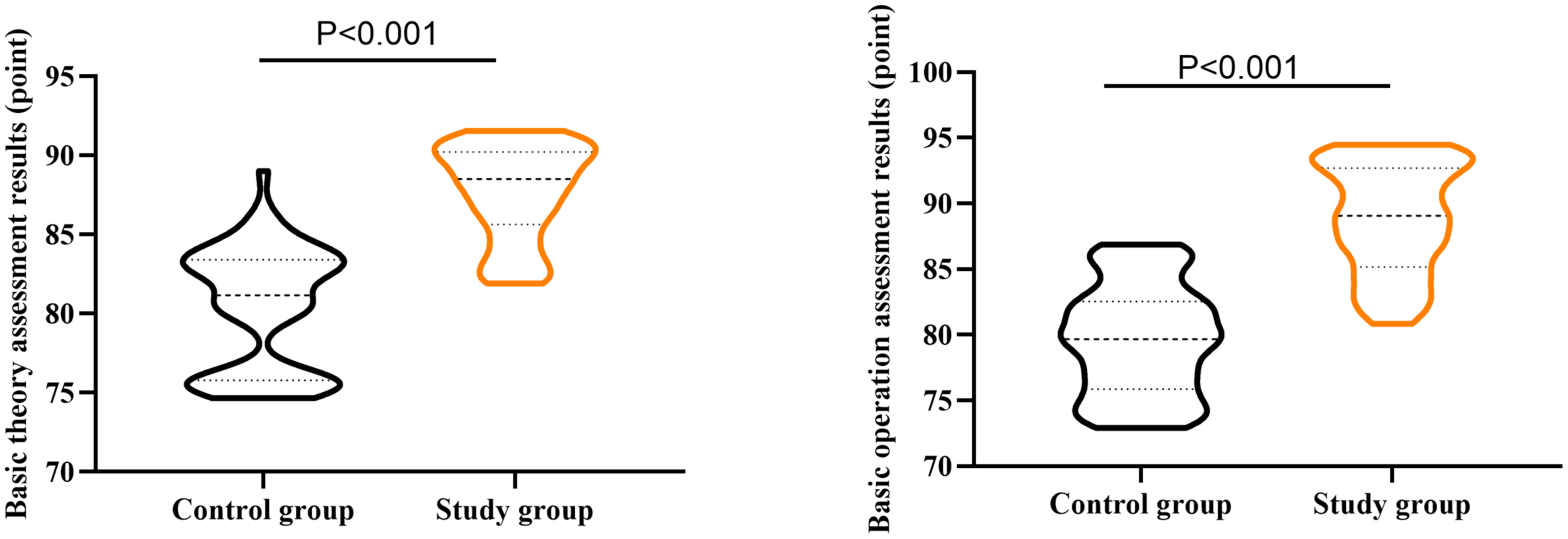
Assessment results in two groups. The *t*-test was used for comparison between the two groups.

As shown in Figure 1, the basic theory and basic operation assessment results in the SG were 87.72 ± 2.97 points and 88.88 ± 4.12 points, respectively, and those in the CG were 80.45 ± 3.84 points and 79.62 ± 4.16 points, respectively. Compared to the CG, the SG had higher basic theory and basic operation assessment results (*t* = 11.106, *p* < 0.001 and *t* = 11.729, *p* < 0.001). These results suggest that the self-made video assessment mode can promote the standardized skills of new nurses after the training.

### Core competence of nurses between the two groups

**Figure 2:**
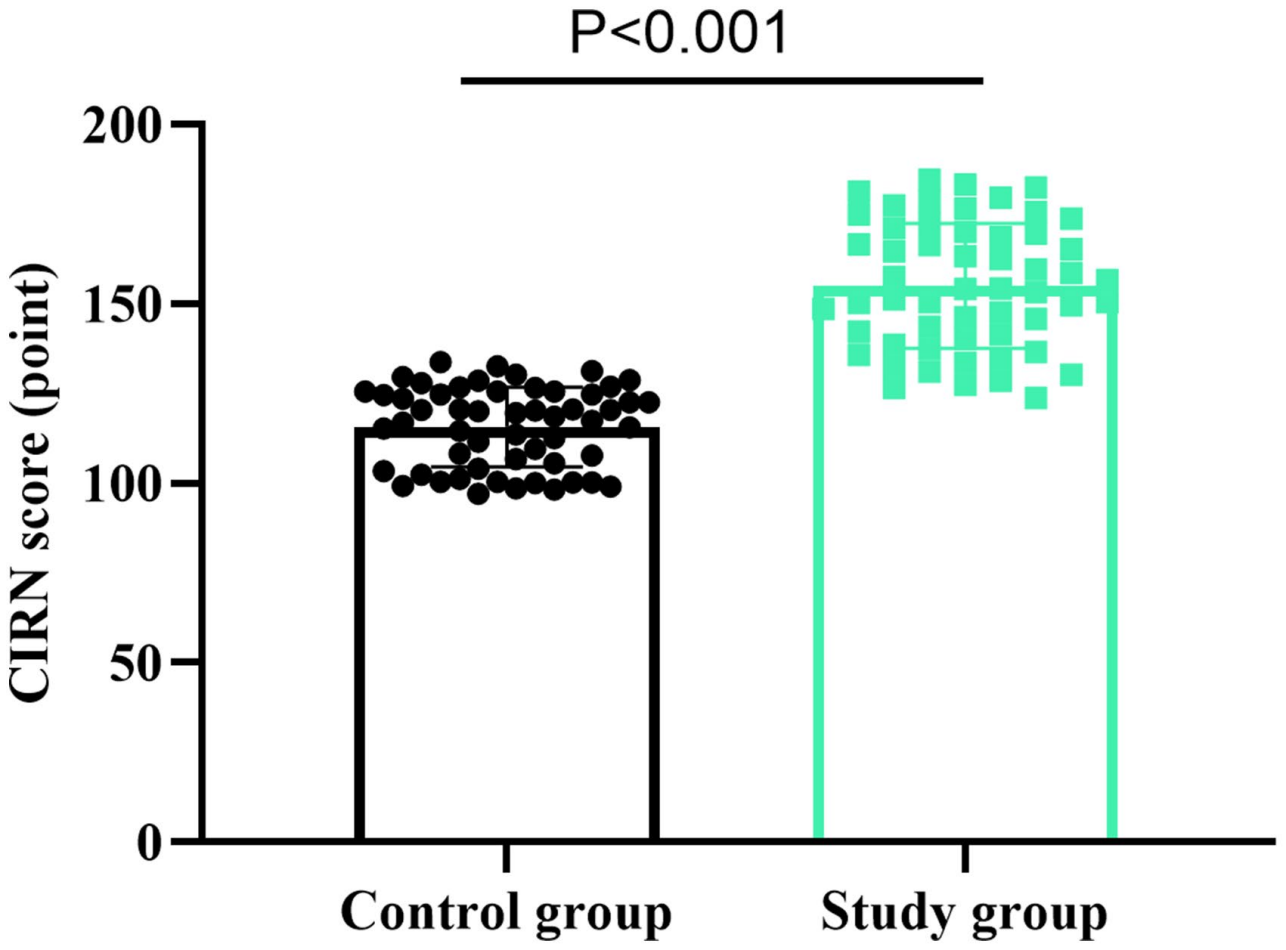
Core competence of nurses in two groups. *t*-test was used for comparison between the two groups.

As shown in Figure 2, the CIRN scores in the SG were 155.01 ± 17.42 points, and those in the CG were 115.69 ± 11.12 points. Compared to the CG, the SG had higher CIRN scores (*t* = 14.109, *p* < 0.001). These results suggest that the self-made video assessment mode can promote the core competence of new nurses after the training.

### Self-efficacy of nurses between the two groups

**Figure 3:**
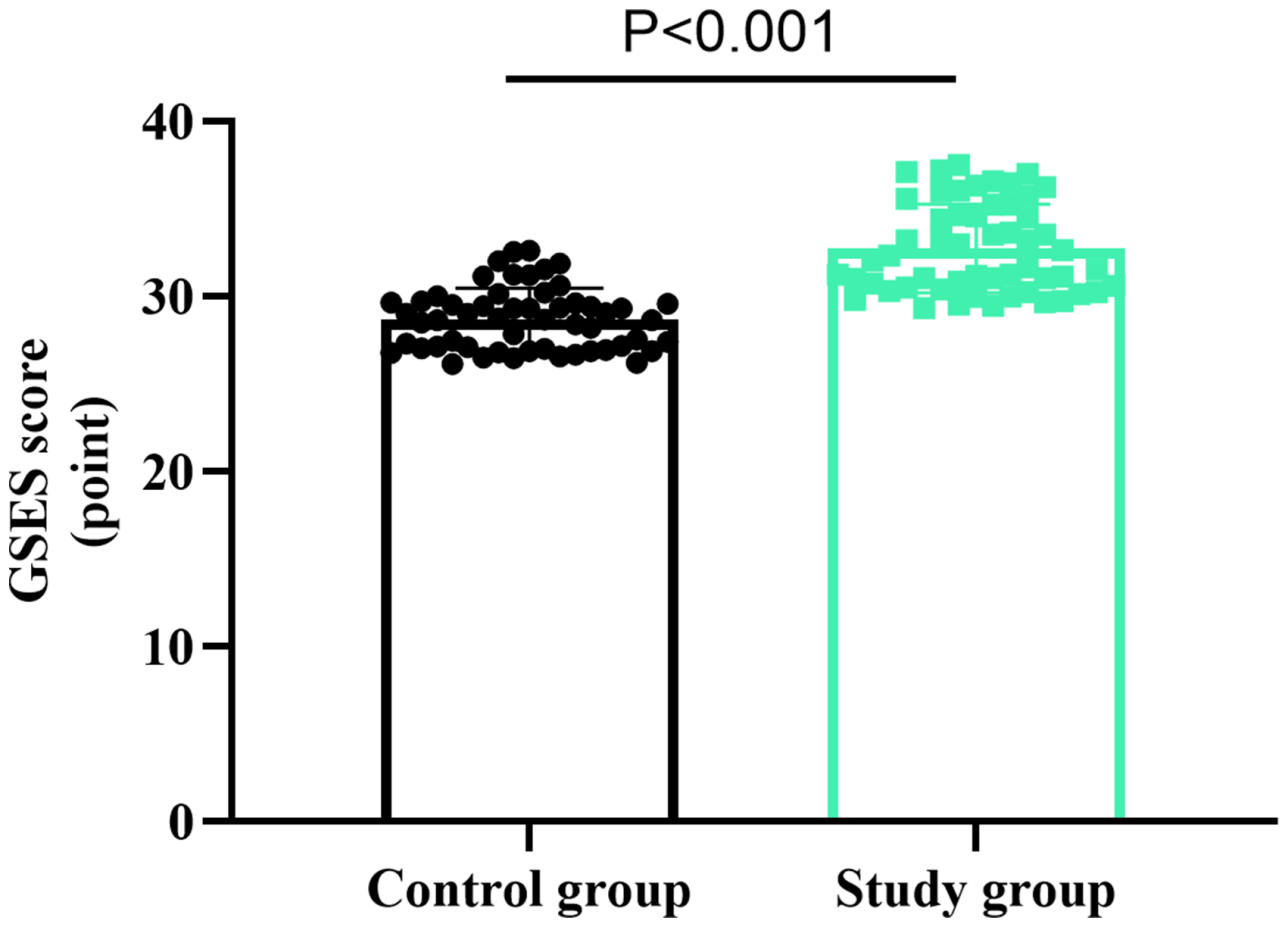
Self-efficacy of nurses in two groups. *t*-test was used for comparison between the two groups.

As shown in Figure 3, the GSES scores in the SG were 32.74 ± 2.53 points, and those in the CG were 27.71 ± 1.75 points. Compared to the CG, the SG had higher GSES scores (*t* = 12.126, *p* < 0.001). These results suggest that the self-made video assessment mode can promote the self-efficacy of new nurses after the training.

### Practice time of nurses between the two groups

**Figure 4:**
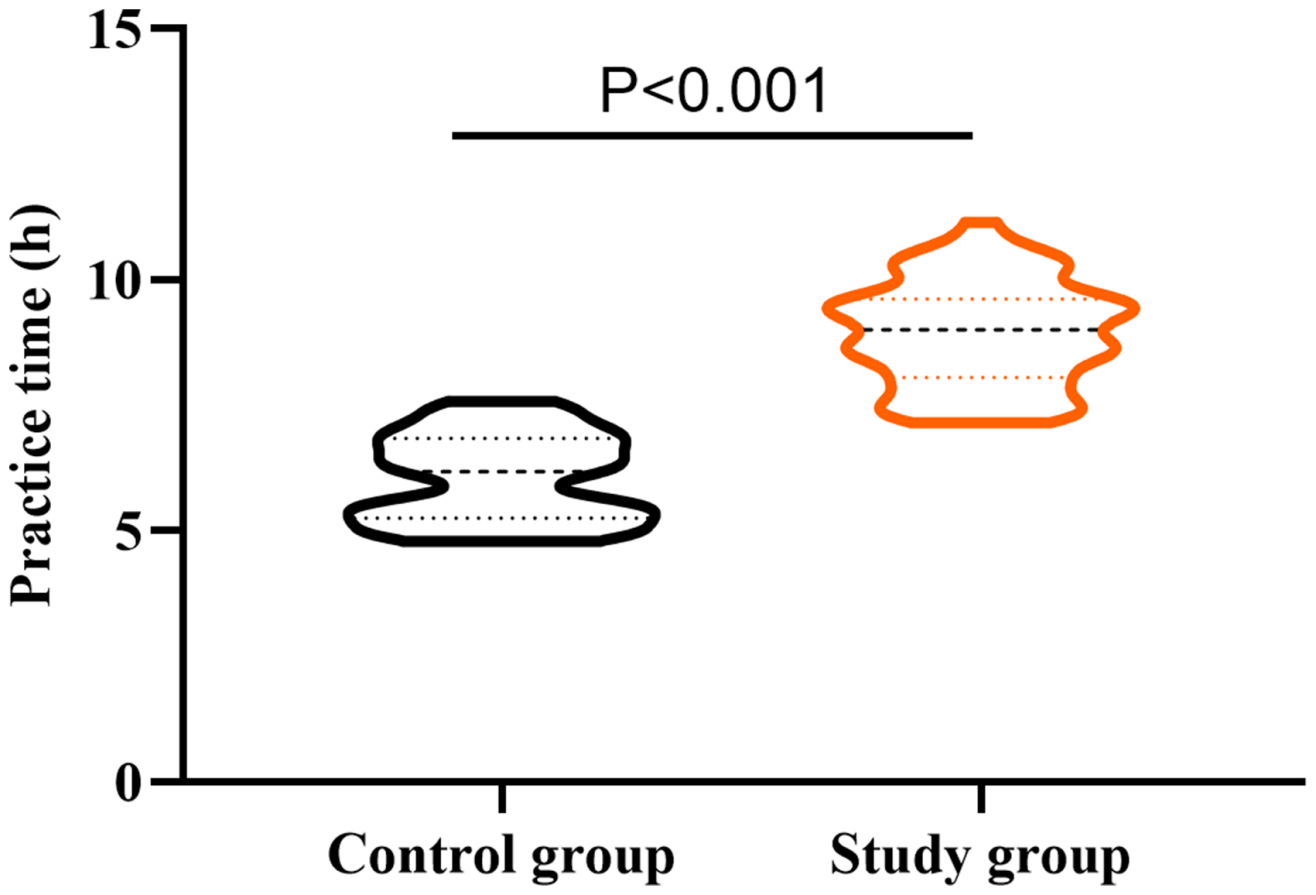
Practice time of nurses in two groups. *t*-test was used for comparison between the two groups.

As shown in Figure 4, the practice time of nurses in the SG was 8.92 ± 1.03 h, and the practice time of nurses in the CG was 6.06 ± 0.86 h. Compared to the CG, the practice time of nurses in the SG was longer (*t* = 15.807, *p* < 0.001). These results suggest that the self-made video assessment mode can increase the practice time of new nurses after the training.

### Satisfaction of nurses between the two groups

As shown in Table 1, the satisfaction of nurses in the SG was 98.18% (54/55), and the satisfaction of nurses in the CG was 85.45% (47/55). Compared to the CG, the satisfaction of nurses in the SG was higher (χ^2^ = 5.930, *p* = 0.015). These results suggest that new nurses are satisfied with the self-made video assessment mode.

**Table 1:** Satisfaction of nurses in two groups.

Groups	Cases	Very satisfied	Satisfied	Dissatisfied	Total satisfaction rate
Control group	55	25	22	8	47 (85.45%)
Study group	55	30	24	1	54 (98.18%)
χ2					5.930
p					0.015

χ^2^ test was used for comparison between the two groups.

## Discussion

Self-made video assessment is a teaching effectiveness evaluation method based on video materials, which can comprehensively examine students’ abilities in analysis, evaluation, and expression (19, 20). Studies have shown that the application of video assessment methods in nursing student evaluations can stimulate students’ autonomous learning ability, enhance practical operation skills, improve students’ skill operation levels, and reduce the workload of teachers (21).

The traditional training evaluation has the drawbacks of a fixed evaluation time arrangement and limited evaluation venues, resulting in low satisfaction of nurses with the evaluation and low evaluation efficiency (22). Face-to-face on-site assessments often cause nurses to be nervous and unable to perform normally, and there is only one chance for the operation, so the examination results often fail to achieve the expected effect (23). This study adopted the self-made video evaluation method, eliminating the tension during on-site operations and the uniqueness of evaluation opportunities. At the same time, nurses can flexibly choose the evaluation time and location, overcoming the limitations of traditional evaluation time and location. In addition, the traditional assessment of operational skills usually only focuses on the nursing operation itself and simply shows the operation process and skills on simulated scenarios and simulated personnel as the basis for scoring, which cannot reflect the comprehensive response ability and actual operation level of the evaluation object from multiple perspectives (24, 25).

This study utilized self-made videos for assessment. Nurses could freely choose between a simulated assessment or an actual clinical assessment. Moreover, before shooting satisfactory videos, nurses would repeatedly practice, which increased the number of practice sessions and, to some extent, enhanced the nurses’ enthusiasm for practice. Due to the repeatability of video assessment, the problems in the assessment could be more easily exposed (26). The reviewers would guide the exposed problems and urge the nurses to make corrections, which helps to improve the nurses’ operational skills (27).

In our study, the results showed that compared to the CG, the SG achieved higher scores in both basic theoretical and operational assessments, and the nurses in the SG spent more time in practice. This indicates that the self-made video assessment model can promote the improvement of nurses’ standardized skills. This indicates that the self-made video assessment model can promote the improvement of nurses’ standardized skills. This is because the self-made video assessment model can enhance nurses’ learning interest, promote their autonomous learning ability, increase their practice time, and significantly improve their assessment scores. This also conforms to the student-centered teaching concept, shifting learning from passive to active (28). Previous studies on assessment models for nursing skills training have mostly focused on the combination of traditional classroom teaching and practical operation assessment, or the integration of online standardized courses and offline assessment (29). Although they have achieved certain results, they lack in-depth guidance and personalized feedback on the nurses’ autonomous learning process. In contrast, the self-created video assessment model in this study not only provides nurses with intuitive and vivid learning resources but, more importantly, through case analysis, operation demonstrations, and interactive sessions, it can adjust the content in a timely manner based on the nurses’ learning progress and feedback, achieving personalized learning guidance. This targeted and flexible approach is unmatched by traditional assessment models. However, for this positive result, we also need to consider other possible explanations. The nurses in the SG might be curious about the new assessment model, thus initially investing more energy and time. This short-term enthusiasm might have contributed to the improvement in scores and practice time, rather than the assessment model itself having a long-term and stable promoting effect.

The core competency of nurses is an essential core ability in the practice of nursing, which should be emphasized by nursing educators, and nursing professionals must possess this ability (30). Self-efficacy mainly refers to the degree of confidence people have when completing a certain task, and this confidence has the characteristic of mastery (31). The assessment of nurses’ self-efficacy has a significant impact on the choice of activities and also determines the attitude during the work process (32). By adjusting self-efficacy and improving the physical and mental health of nurses, the ability of nurses and the quality of nursing work can be effectively enhanced (33). In our study, the results showed that compared to the CG, the SG had higher CIRN scores and GSES scores, indicating that the self-video assessment model can promote the core competence and self-efficacy of nurses, which is consistent with the previous research results (34). In addition, our study also showed that compared to the CG, the nurses in the SG had higher satisfaction, indicating that nurses recognize the self-video assessment model. However, the satisfaction index may also be affected by other factors. The nurses in the experimental group may have higher expectations for the new assessment model, so they gave relatively higher evaluations at the beginning. However, over time, they may discover some potential problems. The current research period may not be sufficient to fully reflect these changes. Moreover, satisfaction may also be affected by factors unrelated to the assessment model, such as team atmosphere and interpersonal relationships.

Our study has some limitations. First, the sample size is relatively small. The research subjects were limited to female nurses newly recruited to our hospital during a specific period. This may cause the research results to be influenced to some extent by the characteristics of the sample and have a certain degree of randomness. Future research can increase the sample size, covering nurses of different genders, from different regions, and at different hospital levels, to improve the reliability and representativeness of the research results. Second, the research period of this study is relatively short. The impact of the self-made video assessment model on the long-term skill retention and career development of nurses has not been deeply explored. Further long-term follow-up studies can be conducted to clarify the long-term effects of this assessment model. Additionally, we did not specifically consider and control the risks of cheating or external assistance that may exist during the self-recording assessment process. The assessment based on unsupervised videos may introduce factors that may affect the internal validity of the research. For example, during the recording process, nurses may receive assistance from others, which may lead to an overestimation of their actual skill levels. Future research should include appropriate monitoring or control measures to address this issue and ensure more reliable assessment results.

## Conclusion

Our study demonstrates that a self-made video assessment approach can enhance nurses’ standardized skills, core competence, and self-efficacy in standardized skills training.

## Data Availability

The datasets presented in this study can be found in online repositories. The names of the repository/repositories and accession number(s) can be found in the article/supplementary material.
